# Dusp4 Contributes to Anesthesia Neurotoxicity via Mediated Neural Differentiation in Primates

**DOI:** 10.3389/fcell.2020.00786

**Published:** 2020-08-19

**Authors:** Jia Yan, Jingjie Li, Yanyong Cheng, Ying Zhang, Zhenning Zhou, Lei Zhang, Hong Jiang

**Affiliations:** ^1^Department of Anesthesiology, Shanghai Ninth People’s Hospital, Shanghai Jiao Tong University School of Medicine, Shanghai, China; ^2^Institute of Neuroscience, Chinese Academy of Sciences, Shanghai, China

**Keywords:** anesthesia, sevoflurane, DUSP4, neural differentiation, primate

## Abstract

**Background:**

Children who are exposed to anesthesia multiple times may undergo cognitive impairment during development. The underlying mechanism has been revealed as anesthesia-induced cognitive deficiency in young rodents and monkeys. However, the molecular mechanism of sevoflurane-induced neural development toxicity is unclear.

**Methods:**

By combining RNA sequencing analysis of macaques’ prefrontal cortex and human neural differentiation, this study investigates the mechanism of sevoflurane-induced neurotoxicity in primates.

**Results:**

The level of dual specificity protein phosphatase 4 (Dusp4) was significantly downregulated in non-human primates after sevoflurane treatment. We further uncovered the dynamical expression of Dusp4 during the human neural differentiation of human embryonic stem cells and found that knockdown of Dusp4 could significantly inhibit human neural differentiation.

**Conclusion:**

This study indicated that Dusp4 is critically involved in the sevoflurane-induced inhibition of neural differentiation in non-human primate and the regulation of human neural differentiation. It also suggested that Dusp4 is a potential therapeutic target for preventing the sevoflurane-induced neurotoxicity in primates.

## Introduction

For young children, the safety of general anesthesia exposure is a critical health issue, which receives widespread attention (Rappaport et al., 2011; Vutskits and Xie, 2016). The U.S. Food and Drug Administration (FDA) issued an official warning that repeated or long-term management of general anesthetic may affect children’s brains development (FDA Safety Announcement December 14, 2016). Until now, three well-known clinical studies evaluate the effects of general anesthesia on neurodevelopment, which are the General Anesthesia compared to Spinal anesthesia (GAS) trial, the Mayo Anesthesia Safety in Kids (MASK) study, and the Pediatric Anesthesia Neuro Development Assessment (PANDA). By using the Behavior Rating Inventory of Executive Function (BRIEF) and the Child Behavior Checklist (CBCL), the results indicate that anesthesia causes specific neurobehavioral changes in infants. What needs to be mentioned is that the MASK study (Warner et al., 2018; Ing and Brambrink, 2019; Zaccariello et al., 2019) assesses the association between multiple general anesthetic exposures and neurodevelopmental deficit, which reveals that the processing speed, fine motor, motor coordination, and visual-motor integration capabilities dampen in multiple but single exposure.

Sevoflurane, the most commonly used anesthetic in children, is reported to induce neurotoxicity and cognitive impairment in non-human primates and rodents (Shen et al., 2013; Zhang et al., 2013; Yi et al., 2016). Aberrant neural differentiation is ascribed to cognitive impairment in young rodents (Cho et al., 2015). Recently, one study even demonstrated that sevoflurane inhibited neural differentiation (Zhang et al., 2019b). Nevertheless, the mechanism is still unknown (Zhang et al., 2015; Wang et al., 2016; Yi et al., 2016; Liu et al., 2020). In addition, in consideration of the different developmental specificity and timing between primates and rodents (Xue et al., 2013), the mechanism of the sevoflurane upon neural differentiation requires further elucidation in primates.

Dual specificity protein phosphatase 4 (Dusp4) is a key gene in neural differentiation (Kim et al., 2015), which is proved to regulate many genes involved in neural differentiation network, such as extracellular signal-regulated kinases (ERKs) (Guan and Butch, 1995; Chu et al., 1996; Ichimanda and Hijiya, 2018), c-Jun N-terminal kinases (JNKs) (Cadalbert et al., 2005), and p38 (Engstrom et al., 2015; Kim et al., 2015; Odaka et al., 2016). Dusp4 is also critical for the endoderm specification (Brown et al., 2008) and cardiac specification (Liu et al., 2007). Irregular expression of Dusp4 might induce carcinoma (Sieben et al., 2005; Venter et al., 2005; Wang et al., 2007; Hasegawa et al., 2008). However, whether sevoflurane-induced neural development toxicity is mediated by dusp4 remains unclear. Thus, in the present study, we aim to explore the effects of sevoflurane on neural differentiation and the underlying mechanisms in primates. By combining the RNA sequencing analysis of macaque’s prefrontal cortex and human neural differentiation, we found that Dusp4 was associated with sevoflurane-induced neurotoxicity. Moreover, sevoflurane treatment–caused Dusp4 downregulation was specifically in non-human primates but mice. Finally, our findings identified that Dusp4 was a target in terms of the prevention and treatment of postoperative cognitive decline in children.

## Materials and Methods

### Rhesus Macaque and Mice Anesthesia

The animal studies were performed according to the guidelines of the Institute of Laboratory Animal Science, Peking Union Medical College, and Chinese Academy of Medical Sciences (Peking, China). The use of rhesus macaque in this research was approved by the Institutional Animal Care and Use Committee (protocol Number XC17001). The control group has three female rhesus macaques, and two female and one male rhesus macaques in the anesthesia group. In this study, the rhesus macaques received 6–8% anesthetic sevoflurane with 100% oxygen for the induction (2–4 min) of the general anesthesia, and then received 2.5–3% sevoflurane and 100% oxygen with endotracheal intubation for 4 h for the maintenance of the general anesthesia. The rhesus macaques received the sevoflurane anesthesia on postnatal day 7 (P7) and then on P21 and P35 days. All the animals returned to their mothers in the cages after the anesthesia. The temperatures of the rhesus macaques were maintained by placing the rhesus macaques in a warm box (37°C). We harvested the prefrontal cortex immediately after the third time of sevoflurane treatment.

C57BL/J6 mice at P6 (Shanghai SLAC Laboratory Animal, Zhangjiang, Shanghai, China) were used in the studies. The animal protocol was approved by the Standing Committee on Animals at Shanghai Ninth People’s Hospital, Shanghai, China. The mice were housed in a temperature- and humidity-controlled environment (20–22°C; 12-h light/dark on a reversed light cycle) with free access to water and food. The mice received sevoflurane anesthesia as described in previous studies (Shen et al., 2013; Lu et al., 2017). The mice in the anesthesia group were exposed to 3% sevoflurane with 60% O_2_ for 2 h daily with 3 days on P6, P7, and P8. The mice in the control group received 60% O_2_. We use a warm box to maintain the rectal temperature of all the mice at 37°C. The prefrontal cortex tissues of mice were harvested at the end of the sevoflurane anesthesia administration.

### Construction of Dusp4 Knockdown HESCs

To downregulate the Dusp4, three Dusp4 shRNA were constructed inside the pGMLV-SC5 vectors. The human embryonic stem cells (hESCs) were cultured in mTeSR 1 medium (EMCELL Technologies, Canada) and Y27632 (1:1000) on Matrigel-coated plates. Cells were digested by Accutase Cell Detachment Solution (Thermo, United States) for passage cultivation. Three shRNAs were mixed to infect the cells. After 48-h infection, mTeSR 1 and 1 μg/ml (final concentration) puromycin were used to screen the transfected hESCs.

The Dusp4-shRNA oligo sequences are as follows:

**Table:** 

**shRNA**	**Oligomeric single-stranded DNA sequence 5′ to 3′**
Control – forward	gatcTGTTCTCCGAACGTGTCACGTTTCAAGAGAACGTGA CACGTTCGGAGAATTTTTTc
Control – reverse	aattgAAAAAATTCTCCGAACGTGTCACGTTCTCTTGAAAC GTGACACGTTCGGAGAACa
shRNA1 – forward	gatccGGAGGCCTTCGAGTTCGTTAATTCAAGAGATTAAC GAACTCGAAGGCCTCCTTTTTTg
shRNA1 – reverse	aattcAAAAAAGGAGGCCTTCGAGTTCGTTAATCTCTTGAA TTAACGAACTCGAAGGCCTCCg
shRNA2 – forward	gatccGCATCACGGCTCTGTTGAATGTTCAAGAGACATTC AACAGAGCCGTGATGCTTTTTTg
shRNA2 – reverse	aattcAAAAAAGCATCACGGCTCTGTTGAATGTCTCTTGAA CATTCAACAGAGCCGTGATGCg
shRNA3 – forward	gatccGCCATAGAGTACATCGATGCCTTCAAGAGAGGCAT CGATGTACTCTATGGCTTTTTTg
shRNA3 – reverse	aattcAAAAAAGCCATAGAGTACATCGATGCCTCTCTTGAA GGCATCGATGTACTCTATGGCg

### Quantitative RT-PCR (qPCR)

The whole RNA was extracted by using RNAiso Plus (TaKaRa, China). cDNA inverse transcription was performed by using cDNA Synthesis Kit (TaKaRa, China). GAPDH is used for reference gene for normalize q-PCR. Detailed qPCR primer information are placed after the references.

Primers for the qPCR detection are listed as follows:

**Table:** 

Pax6	PF: 5′-AACGATAACATACCAAGCGTGT-3′
	PR: 5′-GGTCTGCCCGTTCAACATC-3′
Oct4	PF: 5′-CTTGAATCCCGAATGGAAAGGG-3′
	PR: 5′-GTGTATATCCCAGGGTGATCCTC-3′
Sox2	PF: 5′-TACAGCATGTCCTACTCGCAG-3′
	PR: 5′-GAGGAAGAGGTAACCACAGGG-3′
Nestin	PF: 5′-CTGCTACCCTTGAGACACCTG-3′
	PR: 5′-GGGCTCTGATCTCTGCATCTAC-3′
Tuj1	PF: 5′-TTTGGACATCTCTTCAGGCC-3′
	PR: 5′-TTTCACACTCCTTCCGCAC-3′
Dusp4	PF: 5′-GGCGCTATGAGAGGTTTTCC-3′
	PR: 5′-TGGTCGTGTAGTGGGGTCC-3′
GAPDH	PF: 5′-GGAGCGAGATCCCTCCAAAAT-3′
	PR: 5′-GGCTGTTGTCATACTTCTCATGG-3′

PF, primer forward; PR, primer reverse.

### Neural Differentiation of hESCs *in vitro*

Neural differentiation analysis by using hESCs was performed as described in a previous study (Wattanapanitch et al., 2014). Briefly, the H9 cells were seeded in mTESR 1 medium on Matrigel-coated plates to obtain 80% confluency after seeding. Then the medium was changed to neural induction medium, which contains a 1:1 mixture of DMEM/F12 and neurobasal medium, 1 × N2 supplement, 1 × B27 supplement, 5 μg/ml insulin, 1 mM L-glutamine, 0.1 mM non-essential amino acids, 0.1 mM 2-mercaptoethanol, and supplemented with 1 μM dorsomorphin and 10 μM SB431542. The medium was changed every day for 9 days. Ten days post-differentiation, the NPCs were cultured in the neural maintenance medium, which contains a 1:1 mixture of DMEM/F12 and neurobasal medium, 1 × N2 supplement, 1 × B27 supplement, 5 μg/ml insulin, 1 mM L-glutamine, 0.1 mM non-essential amino acids, 0.1 mM 2-mercaptoethanol, and supplemented with 20 ng/ml bFGF. We also detected the expressions of neural progenitor marker Pax6 on day 12 and the expressions of neuron marker Tju1 on day 25.

### Immunofluorescence Staining

Cells were fixed with 4% paraformaldehyde (Sigma-Aldrich, United States) for 18 min and washed with PBS solution. Then all the cells were treated with 0.2% Triton X-100 (Sigma-Aldrich, United States) for 8 min. Cells were then blocked with 3% bovine serum albumin (BSA) (Sigma-Aldrich, United States) in PBS solution for 1 h. Then incubated cells with primary antibodies, anti-Pax6 antibody (Abcam, ab5790, United States) or anti-TUJ1 antibody (Abcam, ab78078, United States), were diluted in 1% (*w*/*v*) BSA in PBS solution overnight at 4°C. After the incubation, cells were washed with PBS three times and stained with secondary antibodies for 2 h at room temperature.

### Western Blot

Every 50 mg tissues of mouse brain was lysed by the mixture of 0.5 ml RIPA plus 5 μl PMSF on ice. Then the samples were centrifuged at 12,000 rpm for 5 min at 4°C. Cells were lysed by using an SDS buffer (Beyotime, China) to obtain the protein for electrophoresis.

Then all the protein was transferred into PVDF membrane (Bio-Rad, United States). Primary antibodies were used in incubation, including anti-GAPDH (Abcam, ab9485, United States) antibody, anti-DUSP4 antibody (Abcam, ab216576, United States), anti-TUJ1 antibody (Abcam, ab78078, United States), anti-PAX6 antibody (Abcam, ab195045, United States), anti-p-ERK antibody (CST, #8544, United States), anti-ERK antibody (CST, #4695, United States), anti-p-p38 antibody (CST, #4511, United States), anti-p38 antibody (CST, #8690, United States), anti-p-JNK antibody (CST, #9251, United States), and anti-JNK antibody (CST, #9252, United States). Protein expression signaling was visualized through enhanced chemiluminescence (ECL) substrate (Thermo, United States).

### Statistics

The data were presented as mean ± standard deviation (SD). The significance of statistics was analyzed by Student’s *t*-test, one-way ANOVA and two-way ANOVA (^∗^ and # *p* < 0.05, ^∗∗^ and ## *p* < 0.01, ^∗∗∗^ and ### *p* < 0.001). The study employed two-tailed hypothesis and statistically significant *p* values were < 0.05. Each experiment was repeated three times. We used GraphPad (GraphPad Software) to analyze all of the study data.

## Results

### Sevoflurane Decreased the Expression of Dusp4 in Prefrontal Cortex of Rhesus Macaque

**FIGURE 1:**
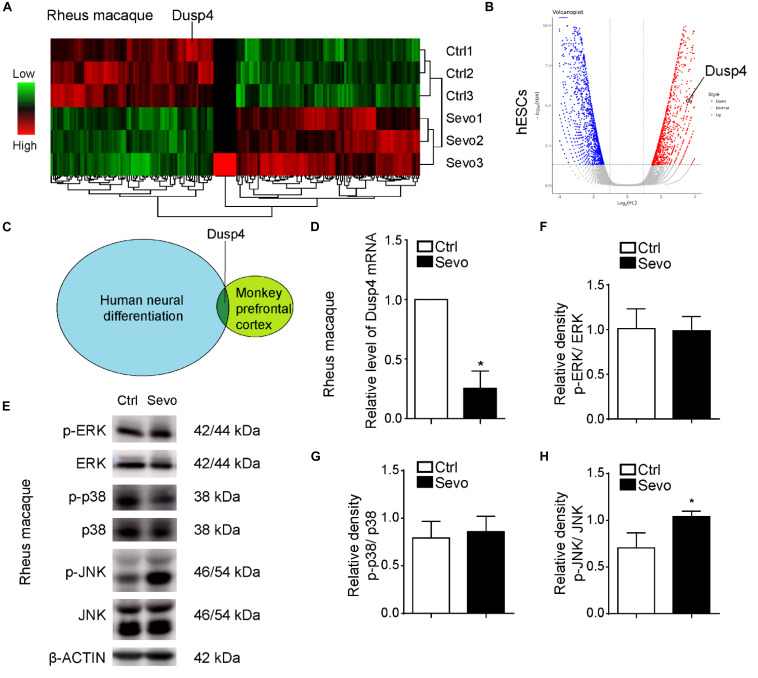
Dusp4 was downregulated by sevoflurane in macaque prefrontal cortex and mediates the human neural differentiation. **(A)** Microarray studies revealed the decrease of Dusp4 expression in brain of macaque treated by sevoflurane compared with the control group. **(B)** Volcano plot of Dusp4 expression upregulated during human neural differentiation (*n* = 3, *p* < 0.05) base on scRNA-seq. **(C)** Dusp4 was downregulated after sevoflurane treatment in rhesus macaques’ prefrontal cortex and upregulated during human neural differentiation. **(D)** qPCR further confirmed the downregulation of Dusp4 expression after sevoflurane treatment in macaque. Data are represented as mean ± SD (*n* = 3). *p < 0.05. **(E)** Steady-state and phosphorylated ERK, p38, and JNK activity was assessed in rhesus macaques’ prefrontal cortex by western blot. Only JNKs, but not p38 or ERK1/2, were consistently phosphorylated in prefrontal cortex of rhesus macaques after sevoflurane exposure. **(F–H)** Qualification of **(E)**. The ratio of phosphor-ERK relative to the total ERK is shown in **(F)**. The ratio of phosphor-p38 relative to the total p38 is shown in **(G)**. **(H)** confirmed the upregulation of phosphor-JNK relative to the total JNK after sevoflurane treatment. Data are represented as mean ± SD (*n* = 3). *p < 0.05.

Alvarado et al. (2017) and us demonstrated that sevoflurane induced myelination damage of the central nervous system and caused behavior changes (e.g., anxiety and visual recognition memory) in infant rhesus macaques (Zhang et al., 2019a). Here, we performed multiple sevoflurane exposures in infant rhesus macaques on postnatal day 7 (P7), P14, and P28 repetitively, with each exposure lasting for 4 h per time as shown in a previous study (Alvarado et al., 2017). After the anesthesia, we collected brain tissues from prefrontal cortex and performed the RNA sequencing to examine the gene expression. As a result, there were 78 upregulated genes and 98 downregulated genes in sevoflurane exposure group (Figure 1A). Neural differentiation is involved in the early state in the infant’s brain development and characterized by rapid structural and functional changes (Vasung et al., 2018). The influence of neural differentiation during early development could result in significant physiological and cognitive impairment (Di Lullo and Kriegstein, 2017; Presumey et al., 2017). Sevoflurane inhibited neural differentiation of mouse embryonic stem cells (ESCs) into neural progenitor cells (NPCs) (Zhang et al., 2019b). To determine the effects of sevoflurane on neural differentiation in primate and clarify the underlying mechanisms, we performed the human neural differentiation with H9 hESCs and detected the whole genome expression by using bulk-RNA sequencing analysis. Interestingly, we combined the results of these two RNA sequencing earlier and found that Dusp4 was downregulated after sevoflurane treatment in rhesus macaque’s prefrontal cortex and upregulated during human neural differentiation (Figures 1B,C). Dusp4 could promote neural differentiation in mice, which is, however, unclear in human neural differentiation (Kim et al., 2015). So, our results indicated its possible function in sevoflurane-induced neurotoxicity. Then we confirmed the downregulation of Dusp4 in prefrontal cortex of rhesus macaques after sevoflurane treatment by qPCR (Figure 1D). To explain the underlying molecular mechanisms, we tested the Dusp4-related classic downstream target genes or signal pathway by using rhesus macaque samples. As a result, we found that JNKs were consistently phosphorylated in prefrontal cortex of rhesus macaques after sevoflurane exposure (Figures 1E–H). Sevoflurane had only effects on JNK phosphorylation in primate (Figures 1E,H). It is consistent with a previous work where induced DUSP4 reduction enhanced c-Jun N-terminal kinase (JNK) activity (Denhez et al., 2019).

### Sevoflurane Has No Effect on the Expression of Dusp4 in Prefrontal Cortex of Mice

**FIGURE 2:**
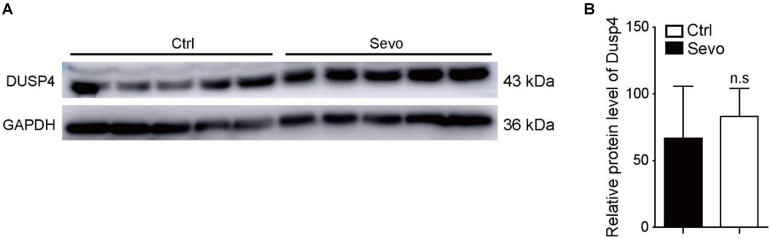
Sevoflurane has no effect on the expression of Dusp4 in prefrontal cortex of mice. **(A)** Western blot indicated that the protein level of Dusp4 was not altered in prefrontal cortex of C57 after sevoflurane exposure. **(B)** Qualification of A showed there is no significant difference of the protein level of Dusp4 between sevoflurane group and control group (*n* = 5).

Anesthetics can induce neuronal apoptosis which causes cognitive impairment in rodents and rhesus macaques (Vutskits and Xie, 2016). So, we exposed mice model to sevoflurane repeatedly, but failed to find similar results. In opposition, the protein level of Dusp4 was not altered in the prefrontal cortex of C57 after sevoflurane exposure (Figures 2A,B). This suggested that mice and rhesus macaques had different gene regulation manners when exposed to sevoflurane. The results indicated that Dusp4 could be the possible sevoflurane-related downstream. In terms of the proximity between human and rhesus macaque, Dusp4 might be still involved in the potential mechanism of sevoflurane-induced neurotoxicity in primates. In addition, it further hinted that the results in rodent model could not fully explain the phenotype in primate due to the differences in species.

### Dusp4 Mediates Neural Differentiation of HESCs Into NPCs

**FIGURE 3:**
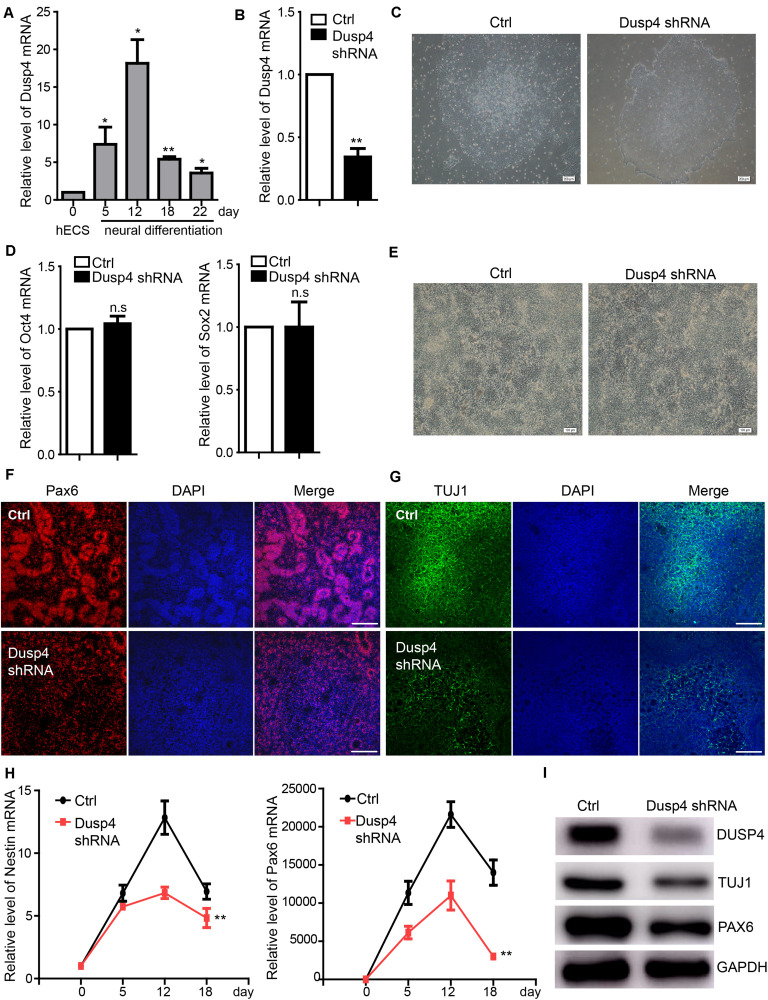
Dusp4 mediates neural differentiation of hESCs. **(A)** qPCR detection of the Dusp4 expression trend during the neural differentiation of hESCs from 0 to 22 days. Data are represented as mean ± SD (*n* = 3). *p < 0.05, **p < 0.01. **(B)** Downregulation of Dusp4 expression by Dusp4 shRNA compared with the control group detected at day 5 during the neural differentiation of hESCs. Data are represented as mean ± SD (*n* = 3). **p < 0.01. **(C)** There was no significant difference in clone formation assay between the Dusp4 knockdown group and control group. Ctrl: hESCs with empty pGMLV-SC5 vector. Scale bar represents 200 μm. **(D)** The results of qPCR showed that there is no significant difference of stemness markers (Oct4 and Sox2) expression between the Dusp4 knockdown group and control group. Data are represented as mean ± SD (*n* = 3). **(E)** Morphology of neural differentiation of two different groups. Scale bar represents 100 μm. **(F)** Immunofluorescence staining of Pax6 indicated that neural differentiation of hESCs was inhibited by Dusp4 knockdown at day 6. Scale bar represents 100 μm. **(G)** Immunofluorescence staining of TUJ1 showed that neurogenesis was further repressed by Dusp4 knockdown at day 28. Scale bar represents 100 μm. **(H)** qPCR analysis showed that dusp4 knockdown repressed the expression of neural progenitor genes (Nestin and Pax6) on days 5, 12, and 18 during the neural differentiation. Data are represented as mean ± SD (*n* = 3). **p < 0.01. **(I)** Results of western blot indicated that the expression of mature neuron gene TUJ1 was downregulated by dusp4 knockdown on day 28.

A previous study showed that Dusp4 regulated retinoic acid–treated neural differentiation in mouse ESCs (Kim et al., 2015). However, whether Dusp4 was involved in the regulation of human neural differentiation remains unknown. Our results showed that Dusp4 was significantly upregulated during the human neural differentiation, especially on day 12 (Figure 3A). Vis-a-vis, sevoflurane downregulated the Dusp4 level which also indicated its potential role in neurotoxicity. To explore the mechanisms further, we downregulated the Dusp4 expression by using three different shRNA mixed viruses (Figure 3B) in H9 hESCs. The results failed to show any influences upon the ability of self-renewal in hESCs (Figures 3C,D). However, the human neural differentiation in hESCs was significantly repressed after downregulation of Dusp4 (Figure 3E). The formation of rosette structure detected by Pax6 was interrupted after downregulating Dusp4, which indicated the repression of neural induction (Figure 3F). The reduction of neuronal class III β-tubulin (Tuj1) (Sullivan and Cleveland, 1986; Caccamo et al., 1989), a marker of mature neuron, further indicated the neurogenesis repression after the downregulation of Dusp4 (Figures 3G,I). In addition, we found that the expression of neural progenitor marker Nestin and Pax6 (Figure 3H) were descended by Dusp4 downregulation. In conclusion, Dusp4 plays an important role in the progress of human neural differentiation.

## Discussion

In the current study, we found that sevoflurane downregulated the Dusp4 expression in prefrontal cortex of rhesus macaque but not in mice. Also, Dusp4-mediated neural differentiation from hESCs into NPCs may be involved in sevoflurane-induced neurotoxicity.

Neural differentiation is ascribed to cognitive impairment in young rodents (Cho et al., 2015). Abnormal neural differentiation results in neurological and psychiatric disorders, serious behavior disorders, and some other nervous system diseases (Goncalves et al., 2016). Neural progenitor cells exist right after infants’ birth for further brain neural development (Rolando and Taylor, 2014). In this study, by combining the RNA sequencing analysis of macaque’s prefrontal cortex and human neural differentiation, we found that Dusp4 may work as a key gene in sevoflurane-induced neurotoxicity. By using the *in vitro* human neural differentiation system, we knocked down the Dusp4 to mimic the Dusp4 downregulation caused by sevoflurane exposure to check the inhibition of neural differentiation. The level of Dusp4 was specifically downregulated after sevoflurane treatment in non-human primates. However, there was no statistical difference of Dusp4 expression in rodents. Our results demonstrated that the mechanisms of neurodevelopment toxicity caused by sevoflurane were different between primate and rodent due to various neurodevelopment. We further uncovered the dynamical expression of Dusp4 during the human neural differentiation by using hESCs. The human neural differentiation could be significantly inhibited after Dusp4 is knocked down.

There are several limitations in the present study. First, we did not monitor the hypoxia and hypotension during general anesthesia in rhesus macaque model. However, the protocol of anesthesia management was the same as in the previous study (Alvarado et al., 2017), which did not show hypoxia and hypotension. Second, the sample size of rhesus macaques (3:3) may not be sufficient enough in the current study; thus, adding the sample size should be performed in the future.

In conclusion, this study indicated that Dusp4 may be involved in the sevoflurane-induced neurotoxicity in non-human primates, which regulated the human neural differentiation in priority.

## Data Availability Statement

The datasets presented in this study can be found in online repositories. The names of the repository/repositories and accession number(s) can be found below: https://www.ncbi.nlm.nih.gov/geo/, GSE148436; https://www.ncbi.nlm.nih.gov/geo/, GSE148437; https://www.ncbi.nlm.nih.gov/geo/, GSE148438.

## Ethics Statement

The animal studies were performed according to the guidelines and regulations of the Institute of Laboratory Animal Science, Peking Union Medical College and Chinese Academy of Medical Science (Beijing, China). Efforts were made to minimize the number of animals in the studies. The use of rhesus macaque in research at the Institute of Laboratory Animal Science was approved by the Institutional Animal Care and Use Committee (Protocol number #XC17001).

## Author Contributions

HJ, LZ, and JY: study concept and design. JL, YC, YZ, and ZZ: acquisition of the data, analysis and interpretation of the data. LZ and JY: draft the manuscript, obtain funding, administrative, technical, and material support. All authors have read and approved the manuscript.

## Conflict of Interest

The authors declare that the research was conducted in the absence of any commercial or financial relationships that could be construed as a potential conflict of interest.
